# Optimized surgical techniques and postoperative care improve survival rates and permit accurate telemetric recording in exercising mice

**DOI:** 10.1186/1746-6148-5-28

**Published:** 2009-08-02

**Authors:** Beat Schuler, Andreas Rettich, Johannes Vogel, Max Gassmann, Margarete Arras

**Affiliations:** 1 University of Zurich, Vetsuisse Faculty, Institute of Veterinary Physiology, and Zurich Center for Integrative Human Physiology (ZIHP), 8057 Zurich, Switzerland; 2 University of Zurich, Vetsuisse Faculty, Institute of Laboratory Animal Science, 8091 Zurich, Switzerland; 3 University Hospital Zurich, Division of Surgical Research, 8091 Zurich, Switzerland

## Abstract

**Background:**

The laboratory mouse is commonly used as a sophisticated model in biomedical research. However, experiments requiring major surgery frequently lead to serious postoperative complications and death, particularly if genetically modified mice with anatomical and physiological abnormalities undergo extensive interventions such as transmitter implantation. Telemetric transmitters are used to study cardiovascular physiology and diseases. Telemetry yields reliable and accurate measurement of blood pressure in the free-roaming, unanaesthetized and unstressed mouse, but data recording is hampered substantially if measurements are made in an exercising mouse. Thus, we aimed to optimize transmitter implantation to improve telemetric signal recording in exercising mice as well as to establish a postoperative care regimen that promotes convalescence and survival of mice after major surgery in general.

**Results:**

We report an optimized telemetric transmitter implantation technique (fixation of the transmitter body on the back of the mouse with stainless steel wires) for subsequent measurement of arterial blood pressure during maximal exercise on a treadmill. This technique was used on normal (wildtype) mice and on transgenic mice with anatomical and physiological abnormalities due to constitutive overexpression of recombinant human erythropoietin. To promote convalescence of the animals after surgery, we established a regimen for postoperative intensive care: pain treatment (flunixine 5 mg/kg bodyweight, subcutaneously, twice per day) and fluid therapy (600 μl, subcutaneously, twice per day) were administrated for 7 days. In addition, warmth and free access to high energy liquid in a drinking bottle were provided for 14 days following transmitter implantation. This regimen led to a substantial decrease in overall morbidity and mortality. The refined postoperative care and surgical technique were particularly successful in genetically modified mice with severely compromised physiological capacities.

**Conclusion:**

Recovery and survival rates of mice after major surgery were significantly improved by careful management of postoperative intensive care regimens including key supportive measures such as pain relief, administration of fluids, and warmth. Furthermore, fixation of the blood pressure transmitter provided constant reliable telemetric recordings in exercising mice.

## Background

Laboratory mice are currently the most common mammalian model for basic and applied biomedical research. Inbred and mutant mice are also universally accepted in the study of inherited human disorders. Thus, mice are widely used for complex investigations requiring additional surgical interventions that are often associated with considerably high levels of morbidity and mortality of the animals. This results mainly from the adverse effects of surgery and trauma, which can be exacerbated by inadequate anaesthesia methodology. The present study focused on refinement strategies aimed at increasing the survival rate in mice after major surgery, using telemetric transmitter implantation as a practical and useful example.

Radiotelemetry is a unique methodology used to measure cardiovascular parameters in unanaesthetized, freely moving small rodents. Using this method, physiological parameters can be efficiently and accurately recorded, allowing reliable and objective results to be established [1-6]. Studying cardiovascular physiology is an important tool in drug development, safety, pharmacology and further research goals [4]. Thus, the ability to record cardiovascular parameters in mice has become an important tool in understanding the response of the cardiovascular system in various experimental approaches [7]. Under certain circumstances, it is necessary to investigate cardiovascular performance under challenging physical conditions. Treadmill exercise tests, which induce cardiovascular stress in order to detect cardiovascular abnormalities that may not be observed at rest, are a commonly used clinical approach in human beings [8]. Metabolic parameters are often determined simultaneously by indirect calorimetry during exercise in man [9]. In contrast to the situation in humans, cardiovascular function and metabolic parameters have been measured only rarely in rodents during submaximal and maximal exercise – the main reasons being the high cost of the necessary equipment and the need for experience in microsurgical techniques for probe implantation. In the case of arterial blood pressure transmitters, the pressure-sensing catheter tip is usually implanted in the thoracic aorta via the left carotid artery, and the transmitter body is placed subcutaneously along the right flank [1]. Thus, the animals have to carry the weight of the transmitter unilaterally. This is particularly uncomfortable during exercise, resulting in abandonment of the exercise test. Moreover, only weak telemetric signals or even no signals at all are obtained during treadmill exercise due to the distance between the transmitter (located on the mouse's flank) and the receiver plate (placed over the treadmill). Furthermore, in spite of advancements in surgical techniques in recent years, morbidity and mortality remain high if telemetric transmitters are implanted in mice. Due to their specific phenotypical characteristics, mice often react with severe impairment of their general condition and symptoms of suffering as a result of the trauma of implantation [10,11]. Thus, even if the surgical procedure appears to be successful, optimized perioperative conditions (e.g. safe and controllable anaesthesia) and intensive postoperative medical care are essential to prevent severe physiological aberrations and death after transmitter implantation. Obviously, such supportive measures are especially important in genetically modified mice with pre-existing bodily abnormalities or compromised compensation potential.

Here we present a detailed systematic intensive care regimen that can be applied after major surgery in mice. The beneficial effects on survival rate were validated in the transgenic mouse line C57BL/6-TgN(PDGFBEPO)321Zbz (tg6), which constitutively overexpresses human erythropoietin (Epo) cDNA, and in corresponding inbred wild type (wt) mice [12,13]. Moreover, we have developed an alternative implantation technique for telemetric blood pressure transmitters, namely to place the transmitter body in the midline of the mouse's back. This approach aimed to minimize the negative impact of transmitter weight on running performance while still allowing telemetric signals to be obtained in a constant and reliable manner during maximum exercise.

## Results

### Standardization of implantation technique

All implantations were carried out by the same experimenter, who was skilled and experienced in microsurgery and implantation of devices in mice for long-term survival experiments. In a preliminary pilot experiment (see below), the implantation methodology (including supportive measures during implantation) was established and practiced until the technique was optimized and highly reproducible.

### Implantation of blood pressure transmitters

The implantations were carried out in a laminar flow hood equipped with a surgical microscope. Aseptic conditions were assured by the use of autoclaved instruments and sterilized materials. Inhalation anaesthesia was initiated and maintained, respectively, with 7–8% and 3.5–4% sevoflurane (Sevorane^®^, Abbot, Cham, Switzerland) in O_2_. During anaesthesia, the animal's eyes were protected with ointment (Vitamine A, Bausch & Lomb, Steinhausen, Switzerland). After shearing of hairs and disinfection of the anterior neck region, a longitudinal skin incision of 1 cm was performed, the connective tissues and muscles were prepared and the left common carotid artery (*Arteria carotis communis sinistra*) was exposed. The artery was ligated with a silk suture (PERMA-Handseide, 6-0, Ethicon, Norderstedt, Germany) at its bifurcation at both the internal and external branches. A second silk suture was placed around the artery at a distance of 4–6 mm caudal of the bifurcation and the blood flow was stopped by retracting the suture. A third silk suture was bound loosely around the artery between the other two. Then, a hole was cut in the artery using fine-bladed scissors and the catheter of a TA11PA-C10 transmitter (DataSciences International, St. Paul, MN, USA) was inserted into the vessel. The middle silk suture was tightened and the retracting, caudal thread was released to allow the tip of the catheter to be advanced into the thoracic aorta. The catheter was then secured in the carotid artery by knotting the silk sutures. The muscles and connective tissues were restored with absorbable sutures (VICRYL 6-0, Ethicon, Norderstedt, Germany). A pocket between the skin and the muscle layers was prepared by blunt dissection with atraumatic scissors. This pocket in the subcutaneous connective tissues reached from the right edge of the skin incision to the back of the mouse. The transmitter body was inserted into the pocket at the right edge of the skin incision and then advanced in a dorsal direction until it was located subcutaneously on the upper back. The transmitter body was then fixed with 3–4 loops of surgical stainless steel sutures (EH 7623G, 3-0; Ethicon, Norderstedt, Germany), which were laid from one side to the other through the skin in the subcutaneous connective tissues underneath the transmitter body. One to two loops of stainless steel wire were placed behind the transmitter body to prevent its dislocation to the lower back. Finally, the skin incision in the neck was closed with absorbable sutures and anaesthesia was stopped. Throughout the entire study, the time required for anaesthesia and implanting the transmitter in each individual was routinely 50–60 min.

### Supportive measures during implantation

The laminar flow work bench on which the mouse was laid during surgery was equipped with a water-bath-heated surface set at 38°C to avoid any drop in the mouse's body temperature due to anaesthesia. When anaesthesia was withdrawn, the conscious animal was allowed to recover for 2–3 hours on the warmed surface (during which time it could move freely under a wire mesh grid) before being transferred back to its home cage.

To support fluid homeostasis during surgery, 1 mL of saline (0.9%) at a temperature of 36°C was injected intraperitoneally shortly after induction of anaesthesia. This was also intended to prevent hypovolemia and hypotension in the case of substantial blood loss from possible bleeding during surgery.

As anti-infective prophylaxis, Sulfadoxin and Trimethoprim were injected intraperitoneally at a dosage of 30 and 6 mg/kg bodyweight, respectively, before starting surgery.

### Analgesics

Either buprenorphine (Temgesic^®^, 0.1 mg/kg body weight; Essex Chemie AG, Lucerne, Switzerland) or flunixine (Biokema Flunixine^®^, 5 mg/kg body weight; Biokema SA, Crissier-Lausanne, Switzerland) was applied. Analgesics were administered subcutaneously twice per day (i.e. every 12 hours) for 7 days; the first injection of analgesics was performed during transmitter implantation.

### Postoperative analgesia and care regimens

All animals recovered within 10–15 min after cessation of inhalation anaesthesia, and showed undisturbed moving behaviour, self grooming, and occasional climbing at 1–2 hours after implantation. All mice were active and in good general condition when transferred to their home cage 2–3 hours after implantation was completed.

Mice in the first two groups were transferred back to standard housing conditions (described below) 2–3 hours after implantation was complete. In the first group, pain was alleviated with buprenorphine (wt: n = 2, tg6: n = 2); in the second group flunixine was used as analgesic (wt: n = 1, tg6: n = 4). No further postoperative measures were applied in these animals. All animals from these two groups were found moribund with severe symptoms of hypothermia, apathy, and exsiccosis at 1–14 days after implantation. The wt mice in the first and second group had a mean survival time of 9 days, whereas the tg6 mice had a mean survival time of 4 days after implantation. Animals treated with the opioid buprenorphine (first group) had a mean survival time of 4 days, whereas treatment with the non-steroidal anti-inflammatory drug flunixine (second group) showed a tendency towards longer survival, with a mean survival time of 7 days.

Necropsy and transmitter explantation both confirmed that the implantation per se was optimal, with no signs of specific complications (e.g., bleeding, necrosis, inflammation, wound dehiscence, or entrapment of legs or teeth in a wire loop). Further systematic pathological investigation revealed no abnormalities and no hints of side effects from the different types of pain killers used. It was assumed that the prolonged deficiency in energy and fluid intake combined with the extended postoperative energy demand and temperature loss were the causes underlying the deterioration in bodily functions and fatal outcome in the first two groups.

The results from the first two groups led us to develop improvements in the postoperative care regimen focussed on resolving the energy and fluid deficiencies and supporting maintenance of normal body temperature.

A third group (wt: *n *= 47, tg6: *n *= 48) received flunixine for pain relief and an additional fluid therapy of 300 μl glucose (5%) and 300 μl saline (0.9%), injected subcutaneously twice per day for 7 days. All analgesic agents including glucose liquid and saline were warmed to body temperature before injection. For this third group, all animal cages were kept on a heating mat during the entire 2-week postoperative period. In addition, unlike the first two groups, the third group had free access to glucose (15%), offered in a second drinking bottle, and to a high-energy wet food (Solid Drink^®^-Energy, Triple A Trading, Tiel, Netherlands). The high-energy food and the glucose-containing bottle were provided in the animals' cages from 2 to 3 days before surgery until 2 weeks afterwards. In addition, O_2 _was piped into the cages for two weeks after implantation.

In contrast to the animals of the first and second group, no animal in the third group showed postoperative complications such as hypothermia, apathy, or exsiccosis. Four tg6 mice developed haematoma in the right neck and shoulder area at 1–2 hours after surgery. Three of these animals could be rescued by re-opening the wound and removing the blood clots, while additional warm saline (1 mL) was injected intraperitoneally. Despite removing the haematoma, one of these animals was sacrificed 6 days after implantation because of repeated bleeding at the implantation site. Finally, 94 out of 95 mice in group 3 were healthy and in good physical condition at the end of the 14-day postoperative period.

**Figure 1:**
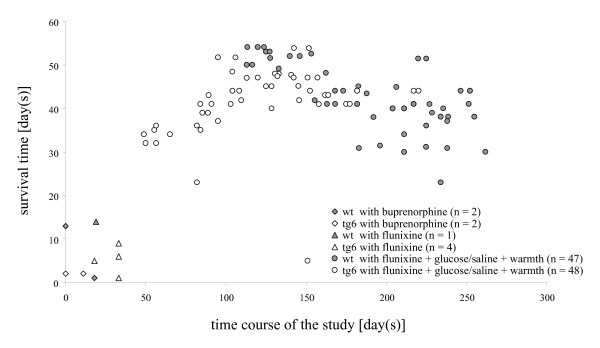
**Postoperative intensive care (administration of analgesics and fluid, warmth) improves long-term survival**. Each value represents a single animal.

Later, three wt mice were sacrificed between 23 and 30 days after implantation as these animals were fighting with their female companions and showed severe skin injuries. Two tg6 mice were sacrificed 23 and 41 days after implantation because the telemetric signals hinted at signs of blood clot formation at the tip of the catheter (i.e. damped blood pressure curves). This was confirmed during necropsy. Despite these limitations, the methodology applied to group 3 was rather successful, as shown by the relationship between postoperative treatment and survival time (Figure 1). The postoperative intensive care regimen established in the third group was associated with survival times of 30–56 days, which was the final endpoint of the study and the point at which the animals were sacrificed after having exercised on the treadmill.

### Survival time vs. body weight

Animals underwent implantation of telemetric transmitters at the age of 4–5 weeks (mean 30.9 days, standard deviation ± 2.7 days). Mice were weighed on the day of implantation (before implantation started) and on the day on which they were sacrificed. Body weight was recorded with a precision balance (PR 2003 Delta Range, Mettler-Toledo AG, Greifensee, Switzerland) especially calibrated for use with moving animals. Body weights were corrected to take into account the weight of the transmitter (1.4 g). All weighing procedures were performed at the same time of day (07:00–08:00 a.m.).

**Figure 2:**
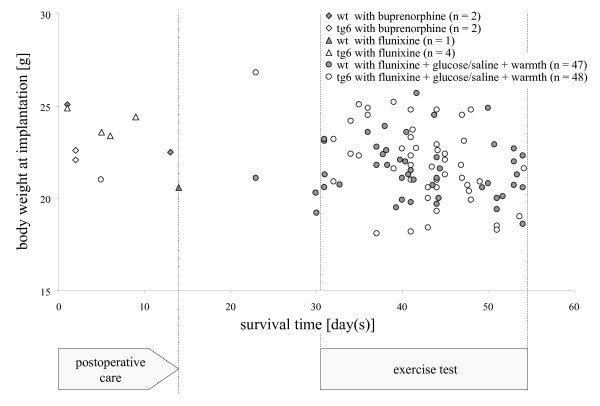
**An individual's body weight at implantation is not correlated with its survival time**. Ten mice with high body weight (21–25 g) did not survive 14 days after implantation; nine of these (triangles and diamonds) represent animals that did not receive postoperative fluid support and warmth. One transgenic mouse (open circles) received postoperative intensive care but was sacrificed 6 days after surgery because of repeated bleeding (probably promoted by the impaired blood coagulation properties of the tg6 mouse line). The remaining 94 mice benefited from postoperative intensive care as they survived and were in a healthy condition at >3 weeks after implantation, regardless of body weight at implantation [even the 14 mice that had particularly low body weight (<20 g) at implantation].

**Figure 3:**
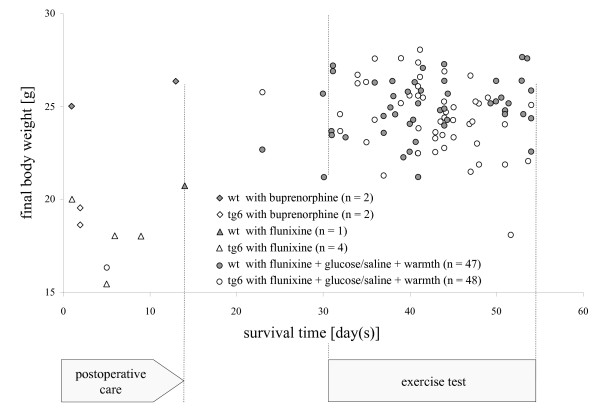
**Final body weight in relation to survival time**. Each value represents a single animal. Compared to the time point of implantation, the transgenic mice (tg6) without postoperative intensive care (open triangles and diamonds) or permanent bleeding, lost body weight, whereas the 3 wildtype (wt) mice (filled triangles and diamonds) without postoperative intensive care maintained or even increased their body weight, although they did not survive 14 days after implantation. Thus, the progress of the individuals' body weight in the 2-week, critical postoperative period could not be taken as a reliable indicator for assessing the quality of convalescence and for anticipating survival. The mean body weight of 94 mice that received postoperative intensive care and survived for more than 3 weeks was increased from 22 g (standard deviation ± 2) at implantation, to a final weight of 25 g (standard deviation ± 2) on average. The long-term gain of body weight confirmed that the animals were healthy and in good bodily condition for exercising on the treadmill, and that they tolerated the transmitter well.

On the day of implantation, body weights ranged from 18 g to 27 g (wt: mean 21.6, standard deviation ± 1.6; tg6: mean 22.2, standard deviation: ± 2.1). There was no correlation between an individuals' body weight at implantation and their survival time. Mice that did not survive more than 2 weeks were around average body weight, or even slightly above-average weight, at implantation (Figure 2). The final body weight of mice that did not survive more than 2 weeks was decreased in the tg6 mice but not in wt animals. Thus, the final body weight was not clearly correlated with survival time. The final body weight of the 94 mice that survived >3 weeks showed that they gained weight over time, as would be expected with increasing age and bodily development (Figure 3).

### Long-term effects of transmitter fixation

**Figure 4:**
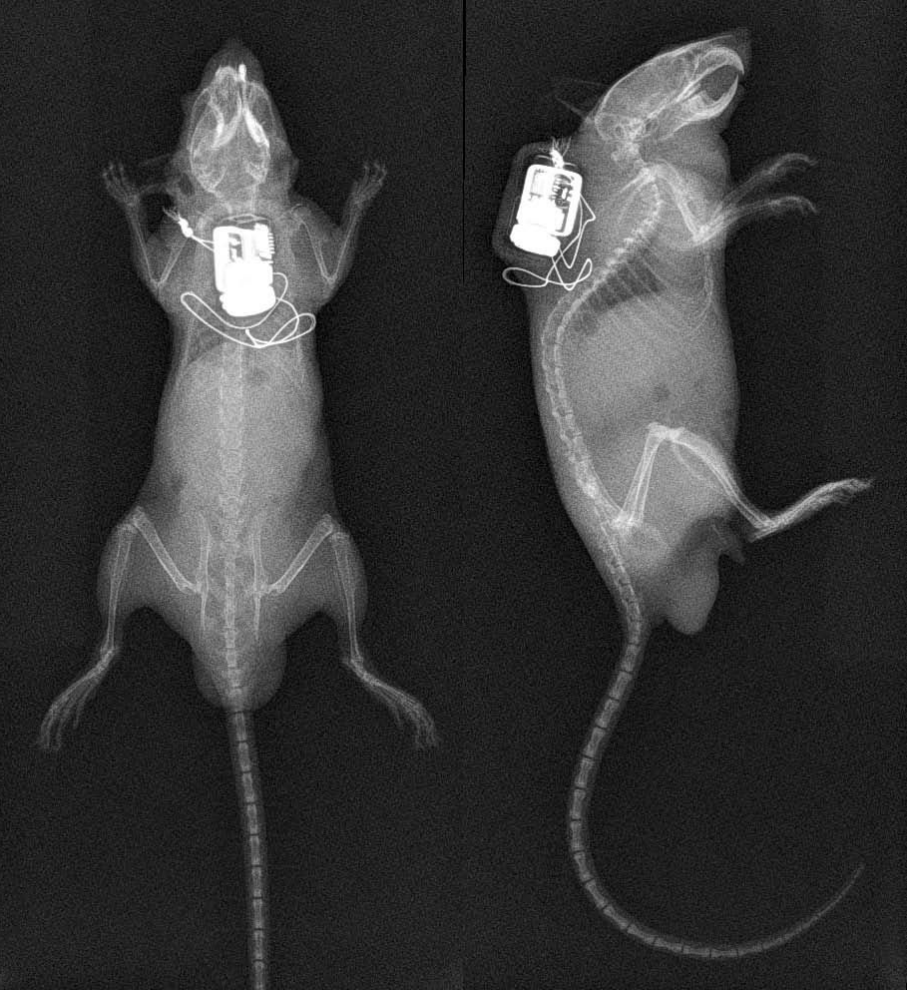
**Radiographs showing the position of the telemetry transmitter from a dorsal (left) and lateral (right) viewpoint**. The transmitter body was placed in the midline of the mouse's back and fixed with surgical stainless steel sutures.

**Figure 5:**
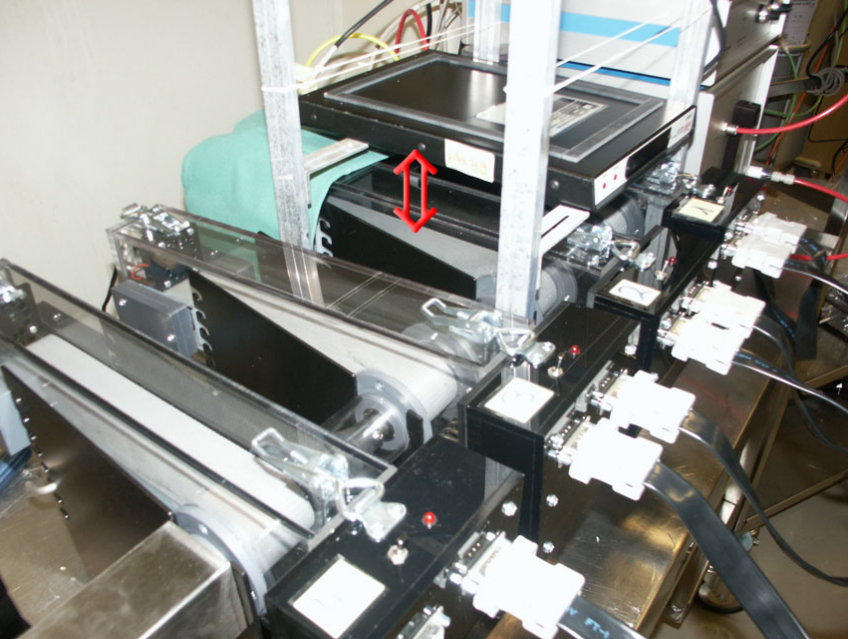
**Treadmill with telemetry receiver plate**. The arrow marks the distance between the running belt of the treadmill and the receiver plate. The secure fixation of the transmitter body on the back of the mouse allowed reliable, constant recording of blood pressure curves from mice during maximal exercise.

In all animals, the transmitter body was fixed in the midline of the mouse's back (Figure 4). In two animals from the third group, the transmitter body had to be re-fixed the day after implantation because it had turned and moved to the upper neck region, where it could hamper the movement of the mouse's head. In general, the mice showed no signs of reduced physical activity or restricted head movement. In contrast to the implantation technique usually used, where the transmitter body hangs loosely at the lateral body side, fixation with stainless steel wires on the animal's back allowed reliable detection of the telemetric signal, as the distance between the telemetric receiver plate and the transmitter body is shortened (Figure 5).

### Telemetric signal verification during maximal exercise

**Figure 6:**
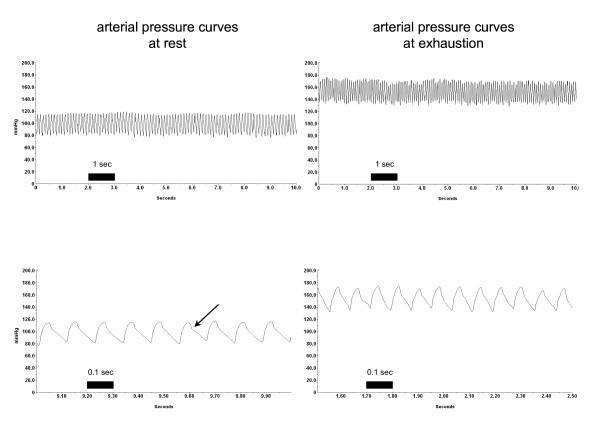
**Representative examples of original data of blood pressure measurements at rest and during maximal exercise (exhaustion) on the treadmill**. The upper row shows 10-second intervals, the lower row represents 1-second intervals cut from the above sample. Typical arterial blood pressure waveforms with a visible dichrotic notch (arrow) were obtained from the same tg6 mouse, with almost no artefacts. At rest, mean values (calculated from 10-second intervals) were as follows: systolic blood pressure 115 [standard deviation (SD) 3.0] mmHg; diastolic blood pressure 86 (SD 2.4) mmHg; heart rate 555 (SD 2.4) beats per minute. At exhaustion, mean values (calculated from 10-second intervals) were: systolic blood pressure 170 (SD 0.9) mmHg; diastolic blood pressure 135 (SD 0.7) mmHg; heart rate 750 (SD 0.01) beats per minute.

The reliability of constant telemetric signaling during maximal exercise was demonstrated in 89 mice at 30–56 days after implantation (Figure 6). The telemetric signal was measured during maximal exercise using a Simplex II metabolic rodent treadmill, fitted with an Oxymax gas analyzer (Columbus Instruments, Columbus, OH, USA). For the maximal incremental exercise test, mice were placed in the exercise chamber and allowed to equilibrate for 30 min. Treadmill activity was initiated at 2.5 m/min and 0° inclination for 10 min, and then increased by 2.5 m/min and 2.5° every 3 min thereafter until exhaustion. Mice were gently encouraged to run for as long as possible by the use of a mild electric grid at the end of the treadmill (0.2 mA, pulse 200 ms, 1 Hz). Exhaustion was defined as the inability to continue regular treadmill running despite a repeated electric stimulus to the mice. Accurate arterial blood pressure curves with minimal artefacts were recorded during maximal exercise on the treadmill in all mice tested.

## Discussion

Our goal of using genetically modified mice with compromised phenotype for performing maximum exercise while obtaining real-time blood pressure measurements was realized by elaborating refined methods for telemetric transmitter implantation and postoperative intensive care.

When telemetric transmitters for measuring blood pressure in mice first became available, a technique for implantation was developed in which the catheter was implanted in the abdominal aorta and the transmitter body was located in the abdominal cavity [14-16]. This implantation method had limited success because it induced thrombosis and embolism at high rates, which finally led to the death of more than half of the mice implanted within 2 days of surgery. Therefore, implantation of the catheter in the thoracic aorta arch via the common carotid artery soon became the method of choice. Because of the limited length of the catheter, the transmitter body had to be placed under the skin of the back, from where it moved usually to the right body wall of the mouse [1,17]. Thus, fixation of the transmitter body with threads attached to the muscles of the animal's back was proposed, but caused difficulties and additional injury, because a second skin incision at the shoulder region was necessary in addition to the one in the anterior neck (used for placement of the catheter) [1]. Thus, it was generally preferred that the transmitter body was introduced via the wound at the anterior neck to the right flank, without any further fixation. This technique, in which the transmitter body hung at the lateral body wall of the mouse, seemed feasible for recordings at rest and during performance of an incremental exercise test, e.g. for about 15 minutes of treadmill running [18]. However, we found in a previous experiment (data not shown) that transmitter signals were either weak, undetectable or disturbed by artefacts during incremental exercise to exhaustion on a treadmill. Moreover, the lateral placement of the transmitter body, which lay in front of the right hind leg, seemed to hamper movement and consequently the exercise performance of the animal. Thus, we inserted the transmitter body through the wound in the neck under the skin of the back, approximately onto the shoulders. Stainless steel wire loops between the transmitter and the connective tissue fixed it in the midline over the axis. Placement of one or two loops behind the transmitter body prevented it from moving to the lower back and avoided the possibility that the catheter tip could be withdrawn from the aortic arch. Any drawbacks of this method were apparent only in the genetically modified mice, in which tissue damage from suturing with wires or injury to subcutaneous vessels when preparing the pocket for the transmitter led to prolonged bleeding due to the impaired blood clotting properties of these transgenic mice (which is a result of the genetic modification). As a consequence, prominent haematoma appeared in the area of the right shoulder and the neck shortly after surgery had been completed. Removing the haematoma by re-opening the wound in the neck and using an intraperitoneal injection of additional saline rescued three out of four of these mice, while one was sacrificed six days after implantation because of repeated bleeding and hypovolemia. Other than these specific transgenic animals, three wt mice were sacrificed before reaching the final time point of the experiment because they sustained serious injuries after fighting with their female cage mates. In these cases, as well as in the two transgenic mice with thrombus formation at the catheter tip, there was no direct relationship between the earlier scarification time point and the new transmitter body implantation technique. Overall, fixation of the transmitter at the upper back of the mouse allowed us to measure reliable telemetric signals at any time point during maximum exercise.

The use of genetically modified mice with phenotypes that hint at deficiencies in physiological and bodily adaption capacities is increasing. Such disabilities in the tg6 mice specifically hampered our surgical efforts in the first part of our study, i.e., the tg6 mice in the first and second group had shorter survival times (average 4 days) compared to the wt, which survived on average 9 days. However, all mice in the first and second groups showed clear symptoms of exsiccosis, hypothermia, and energy deficit, which led to a severely depressed general condition and overall appearance. Subsequently, we introduced additional supportive postoperative measures to improve the recovery of the animals, which led to a high survival rate. We found no relationship between body weight and survival rate, as has been suggested by others [19]; even mice with body weights below 20 g survived implantation without complications if postoperative intensive care was provided. Also, we propose that providing warmth for a prolonged period of 14 days after implantation was beneficial, because this would save energy for the animal, as also proposed by others [20]. In addition, we consider that injection of warmed fluid at 12 hour intervals for seven days was a key intervention supporting survival. Indeed, although this intervention subjects the animals to some additional stress, the positive effect of this therapy appears to far outweigh the risks. Finally, we suggest offering glucose (15%) in a drinking bottle because we observed that the animals consumed the sweet liquid in large amounts postoperatively, thus providing themselves with a continuous source of additional energy and fluid.

Using a combination of the above measures, we were able to overcome the complications and poor success rate of previous implantation methods, since neither wt nor tg6 mice showed the marked symptoms of depressed general condition and health if implantation was followed by intensive care. As a consequence, almost all the mice in group 3 reached the predefined final time point of the study several weeks after implantation. The postoperative intensive care and analgesia regimens described here may be useful for others confronted with similar difficulties when implanting probes or conducting major surgery in mice.

## Conclusion

The present findings show that postoperative care, including pain relief, administration of fluids, and warmth, improves recovery and survival after implantation of probes, which is particularly important in genetically modified mice with impaired bodily capacities. Although our regimen was established for a specialized type of surgery (implantation of telemetric blood pressure transmitters), the optimized postoperative analgesia and intensive care regimen detailed here may be applicable to many other potentially risky or traumatic surgical interventions.

The improvement of the implantation technique by placement and secure subcutaneous mounting of the transmitter body on the upper back of the mouse is crucial to the recording of telemetric signals in the exercising mouse. Thus, even in mice running to exhaustion on the incremental enclosed metabolic treadmill, continuous reliable measurement of cardiovascular function can be achieved.

## Methods

### Ethical review

Each step of the project was approved by the Cantonal Veterinary Department, Zurich, Switzerland, under license number 35/2006. First, preliminary permission for a pilot experiment was approved. In the pilot experiment, the implantation procedure and the perioperative treatment were monitored during site visits by members of the ethical committee and the responsible cantonal veterinarian. Thereafter, the study was fully approved by the authorities. The study was further closely supervised by the ethical committee, the animal welfare officer, and the responsible cantonal veterinarian. In regular site visits, records were controlled, experiments were observed, and modifications to postoperative care were discussed and accepted by the authorities. Written statements from members of the ethical committee and the cantonal veterinary department documented compliance with the ethics of conducting animal experiments in accordance with Swiss Animal Protection Law.

Housing and experimental procedures also conform to the European Convention for the protection of vertebrate animals used for experimental and other scientific purposes (Council of Europe nr.123 Strasbourg 1985) and to the *Guide for the Care and Use of Laboratory Animals *(Institute of Laboratory Animal Resources, National Research Council, National Academy of Sciences, 1996).

### Preliminary pilot experiment

The surgical techniques necessary for the implantation of blood pressure transmitters in inbred mice of variable body weight had been established and standardized prior to the present study. In a preliminary pilot experiment, the anaesthesia and surgery methodologies, including supportive measures during implantation, were optimized and practiced in several wt and tg6 mice (data not shown). At the end of the pilot experiment, the implantation and perioperative procedures were audited and approved by official experts on behalf of the ethical committee.

At the start of the study, the protocol for implantation including supportive measures was standardized as described above. Subsequent postoperative treatment (>1 day after implantation) relied on the experience of implantation of other types of telemetric transmitters in hundreds of mice in our laboratory. Thus, at the time point when the present study began, our routine post-operative care after the animal was transferred back to its cage consisted mainly of administering a pain killer (either an opioid or a non-steroidal anti-inflammatory drug) twice per day.

### Generation and phenotype of erythropoietin-overexpressing mice

The transgenic mice were generated by pronuclear microinjection of the full-length human Epo cDNA driven by the human platelet-derived growth factor B-chain promoter as described previously [12]. The resulting mouse line B6D2-TgN(PDGFBEPO)321Zbz was backcrossed for 10 generations in a pure C57BL/6 background, resulting in the C57BL/6-TgN(PDGFBEPO)321Zbz line, also termed tg6. In general, breeding was performed by mating hemizygous tg6 males to wt C57BL/6 females. As expected, one half of the offspring was hemizygous for the transgene while the other half was wt and served as controls.

Tg6 mice have a Epo plasma level elevated 10- to 12-fold compared to the corresponding controls [12]. Although plasma volume was unaltered, the blood volume of the transgenic animals was nearly doubled. The enormous hematocrit values of up to 0.9 (normal 0.4) strain the heart, and consequently heart weight was markedly increased [21]. On the other hand, blood pressure, heart rate and cardiac output were not altered in tg6 mice. However, bleeding time was significantly prolonged in these transgenic animals [22]. Adaptive mechanisms to excessive erythrocytosis include increased plasma nitric oxide levels and erythrocyte flexibility [12,23]. Recently, it was shown that erythrocyte half-lives in the transgenic animals were 70% lower than in wt controls [24]. Although the tg6 mice grew normally, as a consequence of the adaptive mechanisms, the lifespan of these transgenic mice was one-third that of their wild type siblings [21].

### Animals and standard housing conditions

A total of 50 wt and 54 tg6 male mice were obtained from our in-house breeding colony. The mice were free of the viral, bacterial, and parasitic pathogens listed in the recommendations of the Federation of European Laboratory Animal Science Association [25]. Their health status was monitored by a sentinel program throughout the experiments.

Male tg6 and wt mice were housed individually from 1 week before until 2 weeks after implantation. Later, each male was housed together with an ovariectomized female to avoid the cardiovascular implications of social isolation from long-term single housing as well as the permanent stress and severe injuries that can arise from aggression and fighting – which occur frequently in groups of adult male mice.

All mice were kept in standard rodent cages with autoclaved dust-free sawdust bedding. They were fed a pelleted mouse diet (Kliba No. 3431, Provimi Kliba, Kaiseraugst, Switzerland) ad libitum and had unrestricted access to sterilized drinking water. The light/dark cycle in the room consisted of 12/12 h with artificial light (approx. 40 Lux in the cage) from 3.00 a.m. to 3.00 p.m. The temperature was 21 ± 1°C, with a relative humidity of 50 ± 5%, with 15 complete changes of filtered air per hour (HEPA H 14 filter); the air pressure was controlled at 50 Pa.

## Abbreviations

(Epo): Erythropoietin; (O_2_): oxygen; (tg6): C57BL/6-TgN(PDGFBEPO)321Zbz, transgenic mouse line overexpressing human erythropoietin; (wt): wild type.

## Authors' contributions

BS initiated the study, conducted postoperative intensive care and treadmill exercise, analyzed the data, interpreted the results, and drafted the manuscript.

AR coordinated the study, analyzed the data, prepared the figures, and helped with data acquisition and manuscript preparation.

JV and MG participated in the design of the study and contributed to manuscript revision.

MA implanted the telemetric transmitters, developed the modification of the surgical technique, conceived the postoperative intensive care regimen, and participated in interpretation of data, and drafting and revision of the manuscript. All authors read and approved the final manuscript.
